# Improving the Elastic Response of Tanned Leather by Treatment with a Carboxylic Elastomer

**DOI:** 10.3390/polym16233411

**Published:** 2024-12-04

**Authors:** Daniele Marinai, Cristiana Borchi, Lorenzo Marinai, Gustavo Adrián Defeo, Antonella Manariti, Pierpaolo Minei, Valter Castelvetro, Francesco Ciardelli

**Affiliations:** 1 Kemas s.r.l., Via Sardegna 2, 56029 Santa Croce sull’Arno, Italy; kemas@kemas.eu (D.M.); sicurezza@kemas.eu (C.B.);; 2 CTC Ars Tinctoria s.r.l., Via del Bosco 125, 56029 Santa Croce sull’Arno, Italy; g.defeo@arstinctoria.it; 3 Department of Chemistry and Industrial Chemistry, University of Pisa, Via G. Moruzzi 13, 56124 Pisa, Italy; antonella.manariti@unipi.it (A.M.); ciardelli@spinpet.it (F.C.); 4 Spin-PET s.r.l., V.le Rinaldo Piaggio 32, 56025 Pontedera, Italy; minei@spinpet.it

**Keywords:** styrene-butadiene rubber, elasticized leather, IR analysis, ionomer, modified tanning

## Abstract

The elastic response of chromium-tanned leather was successfully improved by treatment with XSBR, a carboxylated styrene-butadiene copolymer. The carboxylic groups pending from a styrene-butadiene rubber (SBR) backbone were found to promote penetration of the aqueous polymer dispersion into the fibrous tanned leather and participated in pH-reversible physical crosslinking by H-bonding. The different penetrations of XSBR or SBR were investigated using a micro-FTIR cross-sectional analysis from the grain (outer) to the flesh (inner) side of 18 wt% elastomer-treated samples, based on the shaved leather weight. In particular, the profile of the diagnostic out-of-plane =C-H bending of butadiene and styrene units was consistent with a more effective penetration of XSBR. The leather with XSBR showed a comparatively lower elastic modulus of 10–15% and roughly a 10% increase in elongation at the break, indicating better flexibility and shape recovery. Also, the leather was characterized by a 15% higher burst strength. These results suggest the better swelling of the ionomeric XSBR in the initial stage of retanning performed at a pH higher than the isoelectric point of the leather when both the tanned leather and the XSBR ionomer had a negative surface charge. The high pH favored the penetration of XSBR due to a poor attractive interaction with the tanned fibrous leather network. Subsequent processing in an acid bath caused further physical crosslinking through hydrogen bonding between XSBR and the leather.

## 1. Introduction

**Figure 1:**
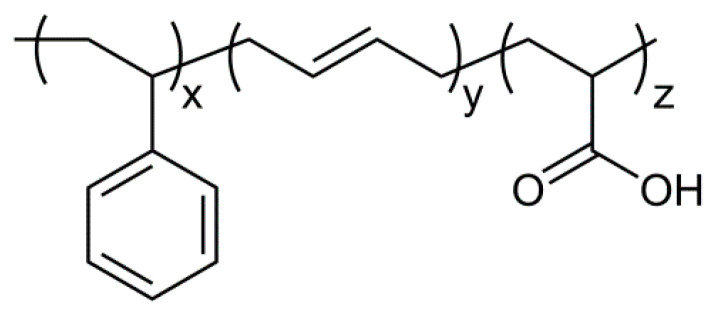
Structure of the XSBR statistic terpolymer (in a typical composition, z is about 3%).

Currently, many carboxylated polymers obtained from post-polymerization modification reactions are commercially available, such as polystyrene (PS) [1], poly(butyl acrylate) [2], etc. Such materials are employed in several applications requiring improved adhesion, compatibility in polymer-based formulations, ion transport capability, etc. Among them, the carboxylated styrene-butadiene copolymer rubber XSBR (Figure 1) has an expanding market [3,4] due to its distinctive characteristics, combining the properties of an elastomeric material with effectiveness in the formation of strong dipolar and hydrogen bonding interactions with other organic polymers, additives, and fillers. Owing to the typically highly improved ultimate properties of the resulting multicomponent materials, XSBR has several industrial applications in, e.g., paper coatings [5], carpet backing [6], gloves [7], water-based paints [8], membranes [9], and polymer-modified mortars [10].

The presence of carboxylic groups in XSBR was found to increase the thickness of the interphase region between the polymer and a second polymeric phase in blends with polar polymers, generally resulting in improved compatibility and, therefore, improved mechanical, rheological, and adhesion properties [11,12]. The strong interfacial interaction of XSBR inorganic fillers and reinforcing components in composites plays a key role in the effectiveness of the synergistic enhancement of the mechanical properties of these materials [13]. Various reinforcing and functional fillers such as multi-walled carbon nanotubes [14], halloysite [15], silica [16], cellulose nanocrystals [17], organically modified boehmite [18], layered silicates [19], chitosan [20], and chitin [21] have been reported in combination with XSBR as compatibilizers or as polymer matrixes.

The formation of a 3D network is one of the main requirements for elastomeric materials. However, such a network does not need to be a covalent one, as effective (although weaker) physical interactions such as in phase-separated rubbery-glassy block copolymers, ionic interactions in ionomers, and suitably located hydrogen bonding interactions may perform the work. In the case of the XSBR pH-dependent ionomer, the hydrogen bonding interactions between carboxyl/carboxylate and hydroxy groups in fillers or other hydrogen bonding groups in binary polymer blends may result in elastomeric properties due to the fillers/second polymer acting as discrete (not point-like) physical crosslinking sites. In XSBR/nano-chitosan (NCS) composites, NCS serves as a multifunctional crosslinker, building a supramolecular hybrid network as well as reinforcement, providing the XSBR matrix with both improved mechanical strength and self-healing behavior [22].

In the present study, the potential of XSBR as an elastomeric reinforcement of tanned leather, characterized by pH-reversible crosslinking behavior through hydrogen bonding [23], was investigated. The pH-dependent physical crosslinking could conveniently provide a self-vulcanizing rubber for the preparation of TPV-type material (TPV: thermoplastic vulcanizate, a type of thermoplastic elastomer that undergoes dynamic vulcanization; that is, mild crosslinking during its manufacturing) or for soft crosslinked materials such as tanned leather. In a reverse-targeted application, collagen-based leather fibers were used to improve the mechanical performance of rubber [21,22]. However, almost no example exists for the addition of rubbery polymers to tanned leather to improve its elastic response. A major obstacle to effective treatments of tanned leather with rubbery polymers is the poor penetration of such generally hydrophobic macromolecules during the retanning process, the latter being performed in an aqueous medium. Neither chemical nor physical crosslinking can easily be achieved once these macromolecules enter the fibrous proteinaceous network of the leather.

**Figure 2:**
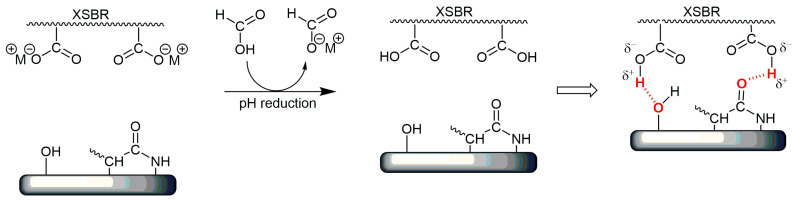
Onset of hydrogen bonding interactions (highlighted in red) between XSBR and the collagen functional groups upon decreasing the pH during the tanning process.

In previous investigations, we found that the elastic response of chromium-tanned leather could be improved by the addition of an XSBR ionomer (about 3% of the carboxylic groups) as a latex dispersion in an aqueous bath treatment [24]. Such an improvement in the mechanical properties was tentatively ascribed to effective penetration and, therefore, a more uniform dispersion of the carboxyl functional elastomer throughout the protein fiber structure of the tanned leather, followed by the development of hydrogen bonding interactions upon decreasing the pH (Figure 2).

## 2. Materials and Methods

### 2.1. Tanned Leather Elastomer Addition

In a typical production process, a load of 100 kg shaved full-grain calf hides with a thickness of about 1 mm was processed in a drum using conventional chrome tanning. In subsequent steps, the tanned leather was then fatliquored, dyed with CI Acid Brown 425, washed with water and then with 0.25% aqueous ammonia to improve wettability, washed again with water, and drained. Afterwards, it was treated with acetic acid to promote the subsequent retanning, washed again with water until a pH above 4.5 was obtained, and retanned (e.g., with the oxazoline-based aldehyde Daplien^®^W 160 product by Kemas s.r.l., Santa Croce sull’Arno, Italy). To further improve the mechanical properties of the retanned leather, 36 kg of Ambrasan DAS^®^ (commercial name of a ~50% solid water-based carboxylated XSBR rubber formulation by Kemas s.r.l.) [24] was added to the same retanning anionic bath at a pH higher than the isoelectric point of the leather substrate, followed after 20 min by the addition of an aqueous solution of formic acid, which lowered the pH down to about 3.5 (lower than the isoelectric point of the leather). The XSBR rubber was a styrene-butadiene-acrylic-acid terpolymer patented product by Kemas s.r.l.; the specific polymer of this investigation was characterized by a 3 wt% acrylic acid content. The obtained rubberized leathers were subsequently drained before performing additional dyeing with an acid dye, treatment with formic acid, washing with water, fatliquoring with an emulsion of natural sulphited oil, further treatment with diluted aqueous formic acid, and washing with water. The obtained leathers were finally extracted from the drum and dried using a multi-step heat treatment that consisted of heating under a light vacuum at a temperature between 40 °C and 45 °C until a residual humidity not exceeding 40% of the leather dry weight was achieved, then they were allowed to dry at room temperature until about 12% humidity (measured using a hygrometer) was reached. Although the typical treatment range was between 2.5% and 27.5%, the samples investigated in this work were treated according to the above procedure and concentrations in which 36 kg of Ambrasan DAS^®^ was used for 100 kg of initial shaved full-grain hides, resulting in a final sample identified as 18% XSBR-treated leather. The same treatment range was used to prepare a reference rubberized leather with conventional (not carboxylated) SBR.

### 2.2. Analytical Techniques

Attenuated total reflectance Fourier transform infrared (ATR-FTIR) spectra were recorded using a Thermo-Fischer Nicolet iS50 FTIR spectrometer (Waltham, MA, USA) interfaced with an ATR ITX accessory equipped with a diamond crystal. Spectra were recorded in the spectral range between 4000 and 650 cm^−1^ using 64 scans and a spectral resolution of 4 cm^−1^. The ATR spectra of XSBR and SBR polymers were recorded on cast films obtained by casting and thoroughly drying the commercial aqueous dispersions at reduced pressure. A micro-ATR analysis on cross-sections of raw and rubber-treated tanned leather was performed with a Thermo Scientific Nicolet iN10 MX integrated FTIR microscope using a slide-on ATR objective equipped with a germanium crystal probe and operated at a 35% pressure strength (maximum pressure 1.5 kg). Samples were cut into 40 µm thick slices at room temperature using a Leica LN22 microtome (Wetzlar, Germany) with a steel blade, and the cross-sectional profile of the tanned leather was analyzed by recording 20–25 spectra along a scan line from the grain to the flesh side. Micro-ATR spectral maps were recorded using a step size of 50 µm for a total of 100 spectra over the selected 500 × 500 µm area. The ATR spectra were recorded using an MCT detector cooled with liquid nitrogen, collecting 64 transients at 4 cm^−1^ resolution in the spectral range between 675 and 4000 cm^−1^. All spectra were normalized using the peak at 1237 cm^−1^ from the C-N stretching and H-N-C bending vibration modes (amide III) [25]. Scanning electron microscopy (SEM) images were recorded at Pontlab s.r.l., Pontedera (Italy) using a Zeiss EVO MA 15 instrument (Carl Zeiss Pvt. Ltd., Cambourne, Cambridge CB23 6DW, UK).

### 2.3. Mechanical Properties

For this research, one bovine leather was divided into enough pieces to obtain samples for the individual application of the different polymers (SBR and XSBR) as well as samples for reference tests. Sampling was conducted using zones of the leather symmetrically positioned at opposite sides with respect to the animal backbone for the two different polymers and along the backbone line for the reference samples to achieve comparative results regardless of the typical fiber structure differences within different areas. Four leather test samples were cut from each specimen, two parallel and two perpendicular to the backbone, and conditioned for 24 h following ISO 2419 recommendations [26]. After measuring the thickness of each sample at three points with a caliper, the mechanical properties were assessed using the following four test methods: ISO 3379:2015 “Determination of distension and strength of surface” (ball burst method) [27]; ISO 3377-1 “Determination of tear load Part 1: Single edge tear” [28]; ISO 3377-2 “Determination of tear load Part 2: Double edge tear” [29]; and ISO 3376 “Determination of the tensile strength, elongation at a specified load and elongation at maximum force of leather” [30]. The distension and the strength of the grain layer (the thin papillary layer of the dermis underneath the epidermis) were evaluated according to ISO 3379:2015 by considering the end point to be the appearance of the first crack as detected with the aid of a magnifying lens. This method emulated mechanical stress during lasting (the stage of fitting the assembled shoe onto a foot-shaped mold or last) in shoe production. Both single-edge and double-edged tear strength tests are performed to verify if leather articles and production processes meet the requirements for given applications (shoes, garments, leather goods, upholstery, etc.); these were performed on samples obtained by cutting two specimens aligned parallel and two additional ones aligned perpendicular, respectively, to the animal backbone. In the ISO 3377-2 method, an elongated hexagonal lozenge is cut off inside the specimen and a dynamometer pulls the two opposite edges, causing the leather to tear. The highest force exerted upon tearing is recorded as the maximum strength. In the more conventional ISO 3376 method [30], a rectangular or dog-bone-shaped leather specimen is clamped to a dynamometer and stretched to measure the elongation at the break, and the corresponding load or tensile strength are expressed in N.

## 3. Results

The retanning of leather using a rubber-based component in the formulation aimed to improve the elasticity and tear strength of the tanned leather. However, a lack of uniform penetration of the rubber, applied as an aqueous emulsion, throughout the leather could result in poor reinforcement of the final leather product. To assess the effectiveness of the penetration and the distribution of the rubber into the tanned leather, a retanning process of chrome-tanned leather was performed in this comparative study with the addition of either one of two rubbery polymers, namely, a carboxylic XSBR ionomer or a conventional SBR. Although a broader range of compositions was prepared and characterized (see the Supplementary Materials, where additional μ-ATR-FTIR spectra taken across the section of leather samples treated with 30 and 55 wt% of either SBR or XSBR are shown in Figures S1 and S2, respectively), the results of the spectroscopic investigation and of the mechanical tests reported hereafter focused on a representative treatment with 18 wt% of the polymer, and the loading referred to the original shaved full-grain leather weight.

### 3.1. FTIR Analysis

**Figure 3:**
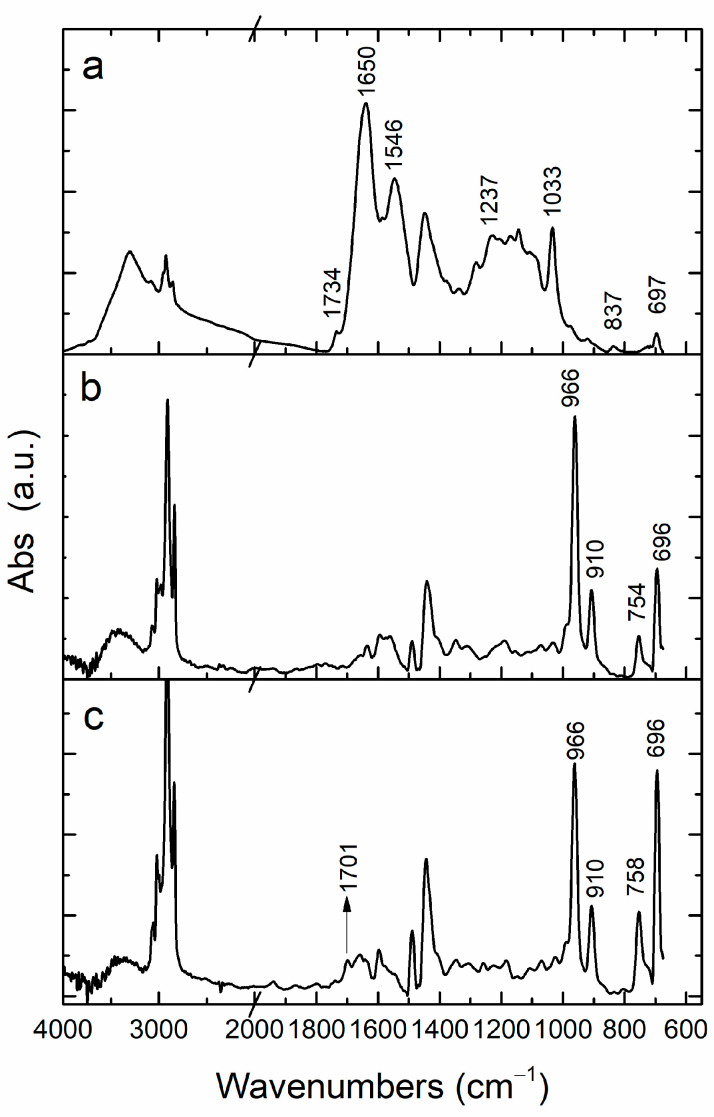
ATR-FTIR spectra of (**a**) untreated tanned leather; (**b**) styrene-butadiene rubber (SBR); (**c**) carboxylated styrene-butadiene rubber (XSBR).

The introduction of carboxylic side groups into the hydrophobic polymer structure was expected to promote its emulsification, its penetration within the tight fibrous network of the tanned leather, and interaction with the proteinaceous collagen fibers, resulting in improved effectiveness compared with the non-carboxylated parent polymer. For this purpose, a simple and sensitive technique was required to assess the effectiveness of the penetration that was capable of discriminating the rubbery polymer from the bulk proteinaceous material along with the acrylic polymer(s) used in the tanning steps. Attenuated total reflectance Fourier-transformed infrared (ATR-FTIR) spectroscopy is a powerful and sensitive tool to assess the presence of specific functional groups on the surface layer of a material. The micro-ATR probe allows mapping of their distribution on a surface or a cross-sectional area, if a cross-section of a sample can be produced. However, monitoring the penetration of carboxylated rubber into the fibrous structure of the tanned leather using the carboxylic group as a marker was quite challenging, even for the FTIR sensitivity, given the low 3% content of carboxylic side groups in the XSBR rubber applied as a leather modifier in this study and also the presence of carboxylic groups in the tanned leather. The ATR-FTIR spectra of the untreated tanned leather and of the two SBR and XSBR rubbers are compared in Figure 3.

The ATR spectrum of untreated tanned leather (Figure 3a) presented characteristic absorptions at 1649 and 1546 cm^−^^1^ from the -HN-C=O stretching (Amide I) and the N-H deformation (Amide II). The weak carbonyl stretching absorption at 1734 cm^−^^1^ could be ascribed to ester groups from lipids (both endogen and added in the fatliquoring step) and to the acrylic copolymer added in the tanning step. In the spectral range below 1000 cm^−^^1^, the weak absorptions due to out-of-plane =C-H deformations at 972, 920, 837, and 697 cm^−^^1^ have been ascribed to vegetable tannins used in the tanning process [31]. In the ATR spectra of SBR and XSBR (Figure 3, profiles b and c), the C-H stretching absorptions of the butadiene and styrene repeat units occurred at 3025 and 3069 cm^−^^1^ (=C-H) and at 2844 and 2915 cm^−^^1^ (main chain methylene). The carbonyl absorption at 1730 cm^−^^1^ from the XSBR rubber (weak carboxylic absorption due to the low concentration) overlapped with the absorptions from the leather matrix (including acrylic copolymers introduced in the tanning step), thus preventing the unambiguous distinction of the two rubbers once applied to the leather (e.g., to assess the possible preferential absorption of one of them). The two rubbers presented additional weaker absorptions at 1638 cm^−^^1^ (vinyl C=C stretching in 1,2-butadiene units), 1601 and 1493 cm^−^^1^ (aromatic ring stretching), and the characteristic peaks for the butadiene units at 966, 910, and 758 cm^−^^1^ from the =C-H deformations of the 1,4-trans, 1,2-vinyl, and 1,4-cis structures, respectively [5]; the peak at 697 cm^−^^1^ could finally be ascribed to the out-of-plane =C-H deformation of styrene units, although it may have also received a contribution from butadiene ones [32,33]. The peaks at 966 and 910 cm^−^^1^ were of diagnostic interest because they could easily be distinguished from the absorptions of the tanned leather matrix. Therefore, they were selected to evaluate the extent of penetration and the distribution of the rubber within the leather. For this purpose, two sampling methods were employed, as described in the next two sections. The first method consisted of slicing the 1 mm thick leather parallel to the sample surface, resulting in three slices; then, the new surfaces, generated at different depths from the original grain and flesh external leather surfaces, were analyzed using ATR-FTIR (or by micro-ATR-FTIR for a more detailed mapping). The second method consisted of cutting the leather perpendicular to the sample surface and then recording an FTIR cross-sectional profile using a micro-ATR probe.

### 3.2. ATR-FTIR Mapping of Internal Layers of Rubber-Treated Tanned Leather

**Figure 4:**
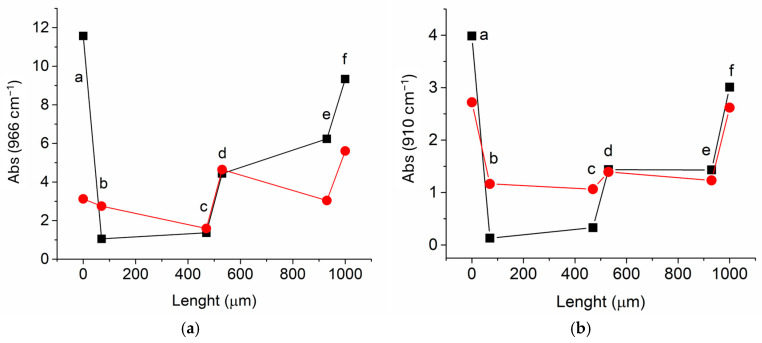
Intensity profile at 966 cm^−1^ (**a**) and at 910 cm^−1^ (**b**) based on the ATR-FTIR analyses of three slices of tanned leather treated with either SBR (black line) or XSBR (red line). The scan line zero-point (length axis) is set on the grain (outer) side. The letters refer to surface maps recorded from layers at the given depth, obtained by slicing the sample parallel to the leather surface (see also the maps in Figure 6 and Figure 7).

ATR-FTIR intensity profiles were obtained by analyzing six surfaces of three slices from each of the two samples treated with XSBR and SBR, respectively. The slices were prepared by cutting the leather parallel to the outer grain surface. The depth profiles at 966 and 910 cm^−^^1^, reported in Figure 4, showed similar trends for each treating polymer, as one would expect because both absorptions were characteristic of the treating polymer with negligible contributions from the leather matrix. On the contrary, clear differences could be observed when comparing the profiles of samples treated with either SBR or XSBR. In the case of the SBR-treated leather, the concentration of the rubber appeared to be higher in the grain and flesh external surfaces (layers a and e in Figure 4) than in the internal layers (layers b and c). In contrast, the intensity profiles of XSBR-treated samples presented a moderate variation, indicating a more uniform distribution of the rubber from the grain surface throughout the bulk and down to the flesh side.

**Figure 5:**
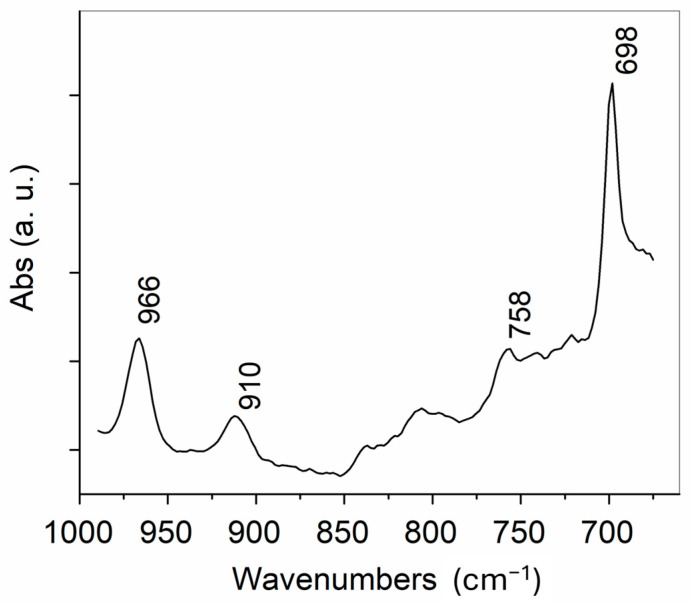
Spectral range comprising absorptions from =C-H deformation modes of styrene and butadiene units, selected to assess the correlation between the reference spectra of the two rubbers (representative spectrum recorded from SBR-treated leather, outer surface, and flesh side).

**Figure 6:**
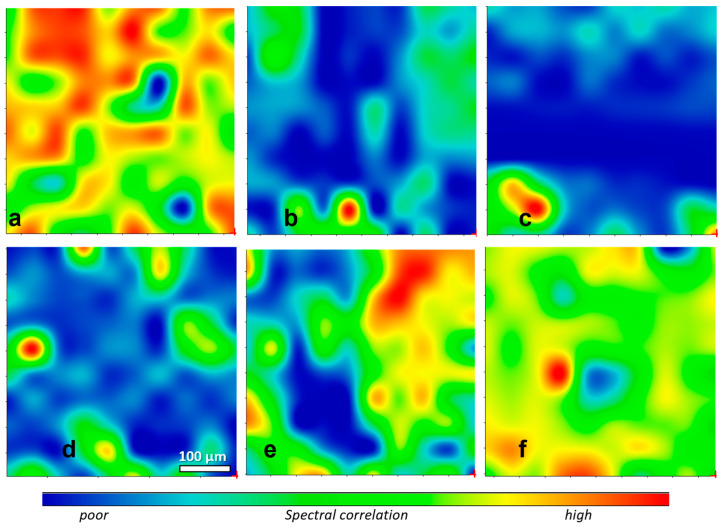
False-color μ-ATR-FTIR correlation maps with SBR as the reference for 1 mm thick SBR-treated leather sliced parallel to the grain surface: (**a**) grain side; (**b**) inner layer at about 70 µm from grain; (**c**) layer at about 470 µm from grain; (**d**) layer at about 530 µm from grain; (**e**) layer at about 70 µm from flesh; (**f**) flesh side. The color range from blue to red indicates increasingly better matches between the μ-ATR-FTIR spectra and that of SBR. A poor correlation indicates that SBR was nearly absent. Map area 500 × 500 μm, scale bar in frame (**d**); spectral range 675–1000 cm^−1^.

**Figure 7:**
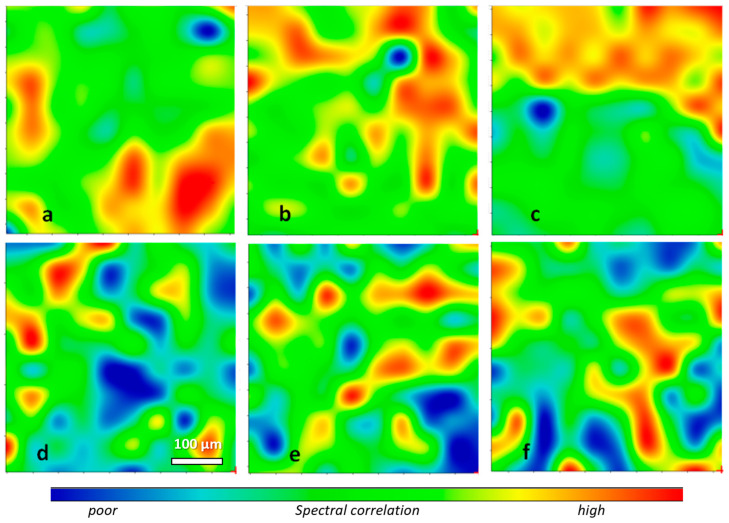
False-color μ-ATR-FTIR correlation maps of XSBR-treated leather slices, recorded as described in Figure 6 (SBR-treated leather): (**a**) grain side; (**b**) inner layer at about 70 µm from grain; (**c**) layer at about 470 µm from grain; (**d**) layer at about 530 µm from grain; (**e**) layer at about 70 µm from flesh; (**f**) flesh side.

The rubber distribution within each of the above surfaces was also evaluated by recording μ-ATR-FTIR maps of 500 × 500 μm areas. These were processed as correlation maps of the recorded μ-ATR-FTIR spectra in the range between 1000 cm^−^^1^ and 675 cm^−^^1^ using the ATR spectrum of pure SBR (Figure 5) as the reference for the correlation, as performed by the software of the FTIR instrument. The selected spectral range comprised absorptions from the =C-H deformation modes of the styrene and butadiene units of both rubbers with negligible contributions from the leather matrix. The correlation maps obtained from slices at different depths, shown in Figure 6 and Figure 7, were in good agreement with the intensity profiles of Figure 4. In the SBR-treated leather, the rubber concentration appeared to be quite high and uniformly distributed on the outer surfaces (Figure 6a,f) with predominant red-green regions showing a good correlation with the SBR spectrum, but were nearly absent in the inner layers across the thickness of the leather (extended blue regions, indicating a poor correlation with the SBR spectrum). This was clearly indicative of poor SBR penetration into the tanned leather. On the contrary, a similar concentration and relatively uniform distribution of the rubber throughout the tested areas and across the leather profile was apparent from the correlation maps of the XSBR-treated leather, confirming the better penetration of the carboxylated rubber.

### 3.3. Distribution of Rubber Within the Tanned Leather Using Micro-ATR-FTIR Depth Profiling

**Figure 8:**
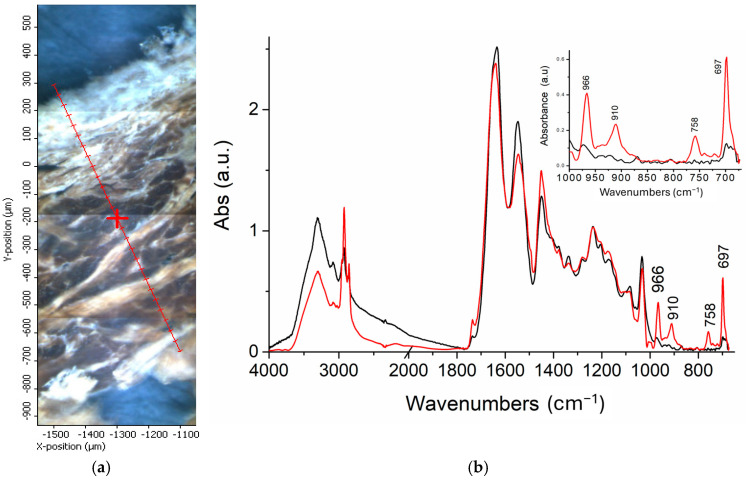
(**a**) Optical micrograph of rubber-treated leather cross-section showing the scan line used for the micro-ATR analyses; (**b**) spectra of SBR-treated (black line) and XSBR-treated (red line) leather samples taken at a position about 650 μm from the grain layer surface, with the magnification of the diagnostic region between 1000 and 670 cm^−1^ shown in the inset.

**Figure 9:**
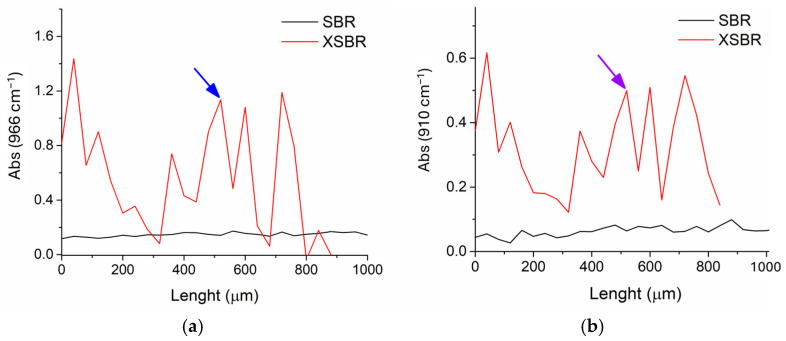
Intensity profile at (**a**) 966 cm^−1^ and (**b**) 910 cm^−1^, for XSBR-treated (red line) and SBR-treated (black line) leather (reported as the ratio of the intensity of the absorption from the tanned leather matrix at 1235 cm^−1^). The scan line zero-point (length axis) is set at the grain (outer) side. The arrows indicate the depth at which the spectra in Figure 3 were recorded.

The penetration effectiveness of the two rubbers was also assessed by recording a micro-ATR (μ-ATR) scan line across sections of SBR and XSBR tanned leather, respectively (Figure 8a). The ATR spectra shown in Figure 8b are representative of the central region of the cross-section, where the largest differences were observed by mapping the layers (Figure 6 and Figure 7). The presence of the characteristic peaks at 966, 910, 758, and 697 cm^−^^1^ from the styrene and butadiene units of the rubbers, clearly observed only in the spectrum of the XSBR-treated leather (red profile in Figure 8b), was consistent with its more effective penetration into the tanned fibrous network. The better penetration of XSBR was highlighted by the μ-ATR intensity profiles along the cross-section of the carboxylated SBR- and XSBR-treated leather samples for absorptions at 966 cm^−^^1^ and 910 cm^−^^1^ from the butadiene units (Figure 9). The intensity of both peaks for the XSBR-treated leather was about three times larger than in the case of the SBR-treated leather, confirming the better penetration of the carboxylated rubber with respect to the not-functionalized one. The large fluctuation in absorbance recorded across the XSBR-treated leather was due to the point-like μ-ATR analysis of the fibrous and highly heterogeneous tanned leather matrix; such fluctuations could also be the result of poor contact between the leather and the ATR probe (Ge crystal) at some points of the cross-sectional surface due to the complex morphology of the analyzed samples.

**Figure 10:**
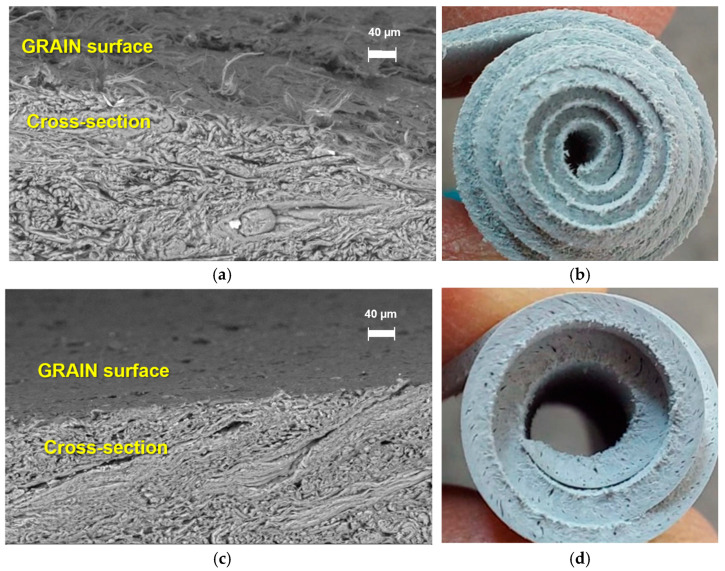
SEM micrographs (**left**) and photograph of the cross-section (**right**) of the leather without an elastomer (**a**,**b**) and treated with XSBR (**c**,**d**).

Scanning electron microscopy (SEM) images were also recorded from the grain surfaces of XSBR-treated and untreated tanned leather. The good penetration of XSBR, applied along with the retanning agents at a pH higher than the isoelectric point of the leather, was confirmed by the improved fullness of the cross-sectional morphology of the treated leather (Figure 10b). It is worth pointing out that the alkaline pH during retanning causes some swelling of the leather and likely facilitated the diffusion of the ionomeric XSBR within the fibrous leather matrix, but it prevented an effective H-bonding interaction between the carboxylate groups of XSBR and the collagen protein of the leather; on the other hand, the soft rubber domains within the fibrous leather structure were expected to turn into elastomeric ones at a final retanning pH ≈ 5 (lower than the isoelectric point of the tanned leather), converting the carboxylate into effective H-bonding carboxylic groups.

### 3.4. Mechanical Response of Rubber-Treated Leathers

The improved mechanical properties of the leather upon application of the carboxylated XSBR within the retanning process were apparent from the results of the mechanical tests, as listed in Table 1, Table 2 and Table 3.

The results of the ball burst test for the determination of the distension and strength of the leather surface (ISO 3379:2015), as shown in Table 1, indicated that treatment with XSBR resulted in about a 25% increase in the elongation at the break compared with the untreated leather. This translated into improved flexibility and higher lastability (i.e., durability in use) of the rubberized leather.

The improved mechanical properties of the leather rubberized with XSBR were further confirmed by the tear strength tests performed according to UNI EN ISO 3377-1 (single-edge tear, also known as “trouser tear” test) and ISO 3377-2 (double-edged tear test). The linear load resistance response to elongation and the tear resistance results, as listed in Table 2, were in good agreement with the results expected for a uniform dispersion of the carboxylated elastomer in the leather matrix, as previously highlighted by the spectroscopic analyses.

To comparatively evaluate the effectiveness of the two rubbers, tensile stress-strain tests were performed according to ISO 3376 on retanned leather samples rubberized with either SBR or XSBR. The results, as reported in Table 3, indicated a slightly lower (about 5%) tensile strength and higher (20%) extensibility, with full deformation recovery upon release of the applied stress, of the XSBR-treated leather compared with the leather treated with SBR. This was a significant and highly desired result when compared with the typical mechanical properties not only of untreated (not rubberized) tanned leather, but also of SBR-treated leather. The highly hydrophobic SBR rubber hardly penetrated the bulk of the leather, as previously shown in the FTIR microscopy analysis of the leather layers, and it could only develop weak hydrophobic interactions, insufficient to convert the SBR rubber into a true elastomer or to achieve the desired physical strengthening of the treated leather.

The enhancement in the mechanical properties of the retanned leather achieved by a relatively small fraction (here, 18 wt%) of elastomer-like XSBR domains and, in particular, the improved elastic response were in agreement with the already-discussed more effective penetration of the carboxylic rubber into the fibrous network structure, along with the excellent adhesive interaction between the elastomeric domains and the tanned leather fibers, further enhanced by the onset of intra- and inter-polymeric pH-reversible H-bonding interactions.

In particular, the improved stretchability of the retanned leather treated with XSBR, as determined by elongation-at-break measurements using different test methods (Table 1 and Table 3), could be ascribed to the combination of a more uniform dispersion of elastomeric XSBR domains in the bulk of the leather than in the case of SBR as well as the effective generation of an H-bonded network involving the carboxylic acid groups of XSBR upon switching the pH to below the isoelectric point of both the leather and ionomer in the last processing stage. Such H-bonding can turn a soft rubber into an elastomeric and highly adhesive material, while the ionomeric nature of the same polymer at a pH higher than the isoelectric point of both the leather and XSBR promoted its dispersion in water and its penetration within the network structure during the leather retanning process.

## 4. Conclusions

The primary objective of this study originated from a successful experimental attempt performed in a technical environment to render elastic the flexible domains present in soft chromium-tanned leather. The approach was simply based on the addition of an aqueous suspension of the carboxylated XSBR elastomer in the tanning reactor during the retanning process, a product innovation subsequently recognized by the release of an international patent. The present study provides scientific support for the molecular interpretation of the observed improvements in the leather properties at a macroscopic level. In particular, the supramolecular structure of the tanned leather, consisting of a crosslinked network of collagen protein fibers, was effectively modified by treatment with XSBR, a styrene-butadiene-acrylic-acid terpolymer characterized by elastic behavior similar to conventional SBR, that was expected to ensure a better interaction with the collagen protein of leather due to the presence of a small (2–3%) amount of carboxylic groups along the macromolecular chain.

Under alkaline conditions, the anionic carboxylate groups of XSBR were found to be critical in promoting the penetration of the ionomer within the fibrous network of tanned leather. The improved penetration of XSBR (vs. the electroneutral SBR) could be ascribed to its improved dispersibility in water and lower tendency to stick to the outer surface of the leather substrate due to the repulsive electrostatic interaction at a pH above the isoelectric point of the collagen fibers. In the subsequent processing step, the lower pH allowed the carboxylate groups to turn into H-bonding carboxylic acid groups. This step was of critical importance; to ensure significant elastic recovery of the deformation, the domains of the XSBR polymer interdispersed within the fibrous material must not only be soft (rubbery, i.e., amorphous and with a low glass transition temperature), but also mildly crosslinked, either physically or chemically, and have a good adhesive interaction with the fibrous leather network. This objective was achieved with the XSBR rubber, which could be turned into a slightly crosslinked elastomeric material through intermolecular H-bonding at an acidic pH.

The presence in the bulk of the leather of soft elastomeric domains, also capable of hydrogen bonding at the interface with the collagen fibers and tanning agents, provided the treated leather with improved stress recovery after deformation, thus limiting the defects produced by fatigue during use.

## 5. Patent

The present work originates from an issued patent to D. Marinai: “Tanning process for producing leather with high elastic properties and leather obtained”, European patent EP3041962.

## Figures and Tables

**Table 1:** Elongation at the break ^1^ of retanned leather in the crust ^2^: influence of XSBR addition.

Sample	Retanned Leather, mm	Retanned Leather + XSBR, mm
1	6.32	9.93
2	8.25	10.11
3	7.25	10.08
Average ± St. Dev.	7.27 ± 0.97	10.04 ± 0.11

^1^ ISO 3379:2015 “Determination of distension and strength of leather surface”. ^2^ In tannery terminology, crust leather is an intermediate dry stage after the wet process (beamhouse, tanning, and retanning) and before finishing.

**Table 2:** Tear resistance of tanned leather: influence of XSBR addition.

Sample	Tear Load: Single-Edge Tear ^1^ (N)		Tear Load: Double-Edge Tear ^2^ (N)	
	Retanned Leather	Retanned Leather + XSBR	Retanned Leather	Retanned Leather + XSBR
Parallel	29.8	36.7	68.3	116.2
Parallel	36.4	55.3	60.2	104.0
Perpendicular	31.2	37.2	62.2	112.3
Perpendicular	34.3	43.5	61.8	110.8
Average ± St. Dev.	32.9 ± 3.0	43.2 ± 8.7	63.1 ± 3.6	110.8 ± 5.1

^1^ ISO 3377-1. ^2^ ISO 3377-2.

**Table 3:** Tensile strength and elongation tests according to ISO 3376 ^1^.

Sample	Thickness (mm)	Tensile Strength ± St. Dev. (N/mm^2^)	Elongation at Break ± St. Dev. (%)
Retanned leather (RL)	1.38	13.4 ± 2.5	47.4 ± 7.4
RL with XSBR	1.42	17.0 ± 1.8	65.7 ± 6.7
RL with SBR	1.35	17.9 ± 2.7	55.7 ± 10.2

^1^ Average and standard deviation of the results from four replicate measurements. Each set of measurements was performed using four leather samples cut from the same leather piece, two parallel and two perpendicular to the backbone.

## Data Availability

The original contributions presented in this study are included in the article/Supplementary Material. Further inquiries can be directed to the corresponding author.

## Supplementary Materials

The following supporting information can be downloaded at: https://www.mdpi.com/article/10.3390/polym16233411/s1. Figure S1: Optical micrograph and line scan recorded by µ-ATR-FTIR on the leather treated with 55% and 30% SBR; Figure S2: Optical micrograph and line scan recorded by µ-ATR-FTIR on the leather treated with 55% and 30% XSBR.
