# Supplementary material for: Improving the Elastic Response of Tanned Leather by Treatment with a Carboxylic Elastomer
Source: Polymers (Basel). 2024 Dec 4;16(23):3411. doi: 10.3390/polym16233411 (PMC11644079; doi:10.3390/polym16233411)
Supplement: Supplementary file 1 [file polymers-16-03411-s001.zip › polymers-3316305-supplementary.pdf]

Supplementary Material

Improving the elastic response of tanned leather by treatment with a carboxylic elastomer

Daniele Marinai [1], Cristiana Borchi [1], Lorenzo Marinai [1], Gustavo Adrián Defeo [2], Antonella Manariti [3], Pierpaolo Minei [4], Valter Castelvetro [3,4,*], Francesco Ciardelli [3,4]

1 KEMAS s.r.l., Santa Croce sull'Arno (PI), Italy
2 CTC Ars Tinctoria s.r.l., Santa Croce sull'Arno (PI), Italy
3 Department of Chemistry and Industrial Chemistry, University of Pisa, Pisa, Italy
4 Spin-PET s.r.l., Pontedera (PI), Italy
* Corresponding author: Valter Castelvetro; email: valter.castelvetro@unipi.it

Characterization of leathers treated with the SBR and XSBR rubbers, respectively, at the concentrations of 30 wt% and 55 wt%.

The line scan recorded by μ-ATR-FTIR on the section of leather samples treated with 55 and 30 %, respectively, of the two rubbers are reported in figure S1 for the SBR-treated leathers and in figure S2 for the XSBR-treated ones. By inspection of the spectral range between 1000 and 600 cm^{-1} it is apparent that SBR does not penetrate inside the leather even at concentrations higher than the 18% discussed in detail in the parent main paper.

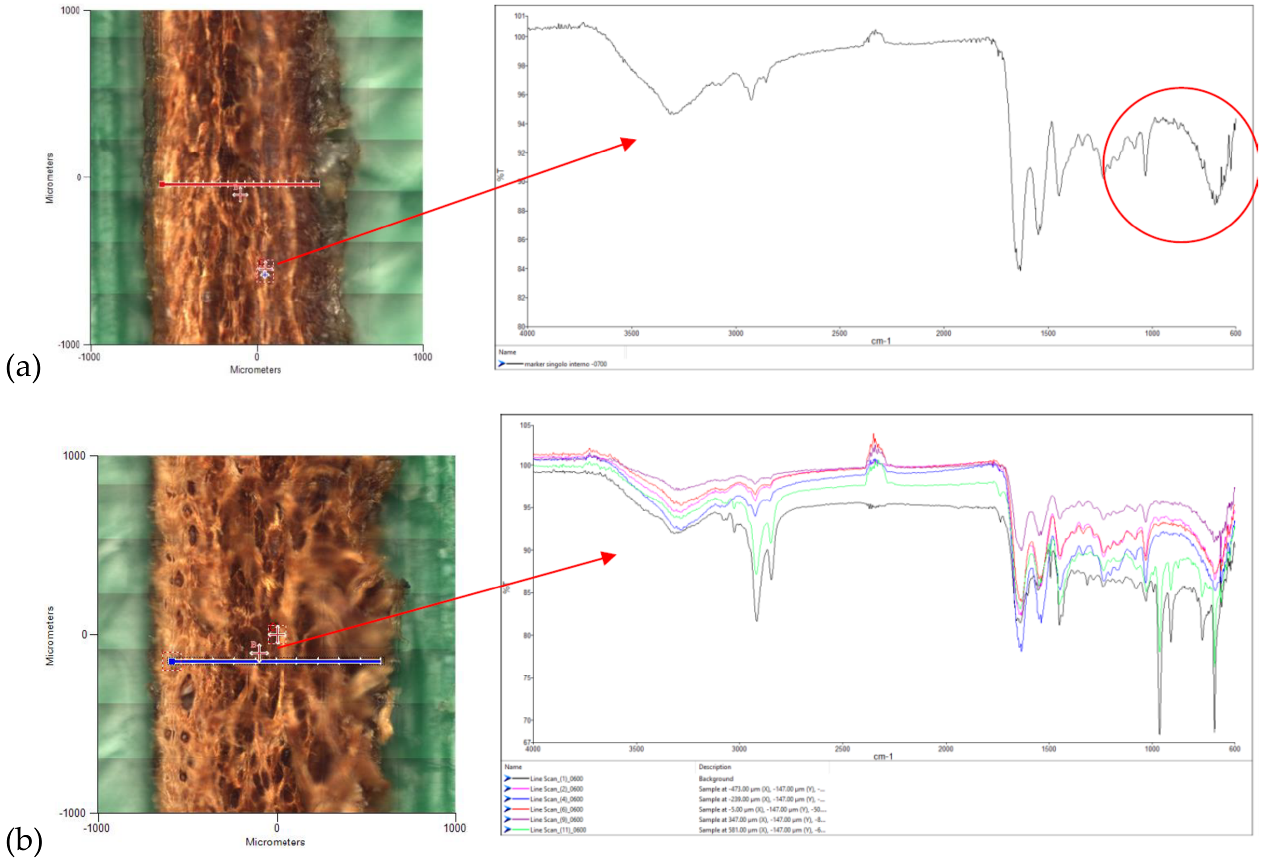

Figure S1. Optical micrograph (left) and line scan recorded by μ-ATR-FTIR on the leather treated with 55 % (a) and 30 % (b) SBR.

(a)

(b)

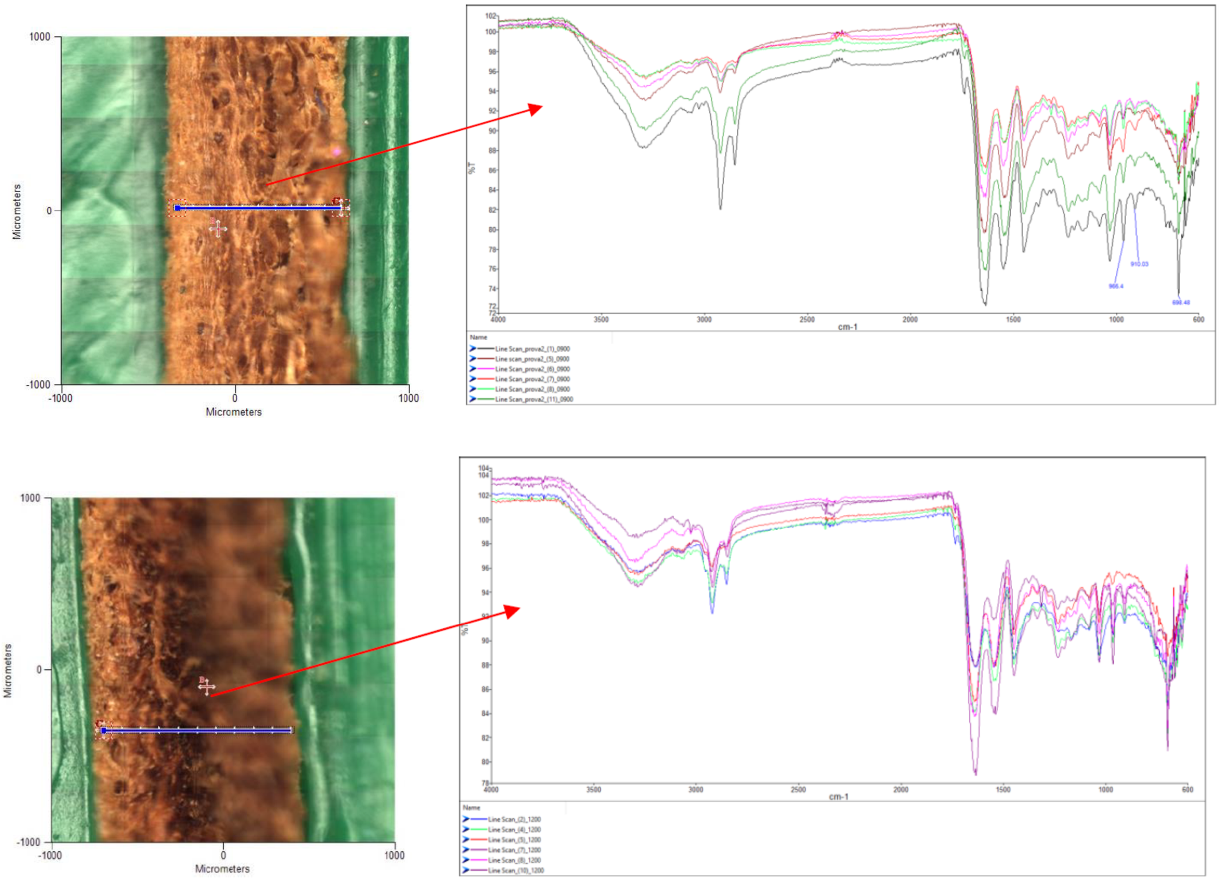

Figure S2. Optical micrograph (left) and line scan recorded by μ-ATR-FTIR on the leather treated with 55 % (a) and 30 % (b) XSBR.
